# Neonatal outcomes and congenital anomalies in pregnancies affected by hypothyroidism

**DOI:** 10.1080/07853890.2021.1970798

**Published:** 2021-09-08

**Authors:** Zareen Kiran, Aisha Sheikh, Khadija Nuzhat Humayun, Najmul Islam

**Affiliations:** a Section of Endocrinology, Department of Medicine, Aga Khan University Hospital, Karachi, Pakistan; b Clinical Fellowship in Paediatric Endocrinology, Paediatrics & Child Health, Aga Khan University Hospital, Karachi, Pakistan

**Keywords:** Hypothyroidism, congenital anomalies, neonatal, outcomes, maternal

## Abstract

**Background:**

Maternal hypothyroidism has been reported to have concerns over neonatal outcomes, not only in the context of neurocognitive development but also in the short term as birth weight and neonatal jaundice.

**Patients and methods:**

We conducted a cross-sectional retrospective study on 638 cases who delivered live births in the Aga Khan University Hospital after ethical approval. Data were collected on hypothyroid pregnant females who were diagnosed before conception or during their antenatal visits during the year 2008–2016. Neonatal outcomes were noted for birth weight, maturity, and neonatal jaundice, neonatal hypothyroidism, neonatal respiratory distress syndrome, sepsis, hypocalcaemia, congenital anomalies, need for intensive care admission, and neonatal death. Subgroup analysis was performed on the timing of diagnosis of maternal hypothyroidism. Data analysis was performed on Statistical Package for the Social Sciences version 20.0.

**Results:**

Neonatal jaundice was the most common neonatal outcome (37.6%) in our cohort of 662 live births. Nearly 15% required intensive care unit admission, however, neonatal death was very rare. The most common clinically significant congenital anomalies were cardiovascular defects, whereas Mongolian spots were the commonest congenital condition to report. There is a statistically significant association between low birth weight (OR 1.86, 95% CI 1.0–3.2, *p* ≤ 0.05) and congenital anomalies (OR 2.39, 95% CI 1.4–4.0, *p* ≤ 0.05) with women diagnosed with hypothyroidism before pregnancy.

**Conclusion:**

We report the neonatal outcomes and spectrum of congenital anomalies of hypothyroid pregnancies diagnosed before and during conception for the first time from the region of Pakistan.

**KEY MESSAGE**
- Overall, none of the neonates of hypothyroid pregnancies developed congenital hypothyroidism.
- Cardiovascular defects in these neonates imply extensive screening and monitoring during pregnancy.
- Low birth weight and congenital anomalies are associated with the timings of diagnosis of hypothyroidism in pregnancy.

## Introduction

Overt hypothyroidism occurs in 2–3% of pregnancies [1] and is well reported to be associated with complications including gestational hypertension (GH) and pre-eclampsia [2–4]. Maternal hypothyroidism also has a number of adverse neonatal outcomes such as low birth weight and preterm birth [5,6] and impaired neurocognitive development in early life of the child [7]. Thyroid hormones appear to have their most profound effect on the terminal stages of brain differentiation, including synaptogenesis, growth of dendrites and axons, myelination and neuronal migration [8]. The timing of vulnerability of brain to iodine as well as thyroid hormone deficiency is within the first 12 weeks of pregnancy until the baby's thyroid begins to produce its own thyroid hormone. Therefore, iodine and thyroid supplementation in the third trimester or during neonatal life doesn't improve neurological outcome [4]. In a case–control study published in 1999, researchers found larger deficit in tests related to intelligence, attention and school performance among children of untreated hypothyroid mothers when compared to children belonging to mothers of the controlled group [9]. Hence if left untreated, hypothyroid pregnancies results in birth of neurological cretin (characterized by neurological, cognitive impairment etc.) [10,11]. Subclinical hypothyroidism has an estimated prevalence rate of 2–5% of all cases [12,13] and is associated with preterm birth [14]. Early placental development by trophoblastic proliferation and invasion requires critical role of thyroid hormones [7,13]. An important risk factor for preterm delivery would be failure of adequate placentation which may result from maternal thyroid deficiency [6,15,16]. Similar mechanism is contributing towards low APGAR (Appearance, Pulse, Grimace, Activity, and Respiration) score and placental abruption leading to stillbirth [3]. Maternal hypothyroidism has also been associated with several congenital anomalies [17,18]. Studies are still difficult to validate strong association of either uncontrolled maternal hypothyroidism or use of levothyroxine drug during pregnancy as a significant risk factor behind these congenital defects or adverse neonatal outcomes [19–21].

We therefore aim to report the neonatal outcomes and congenital anomalies of pregnancies affected by hypothyroidism from a tertiary care centre for the first time from the region of Pakistan.

## Materials and methods

### Ethics approval and consent to participate

The study was approved by The Aga Khan University’s ethical review committee (ERC number: 3977-Med-ERC-15). The study was performed under Helsinki’s ethical principle. To preserve confidentiality, we coded each patient and removed their original identifications. Informed consent was based on hospital consent policy at the time of admission or clinic visit. Moreover, the principle of Justice, Beneficence and Non-Maleficence was maintained and followed throughout the conduct of this study.

### Patient selection

We conducted a cross-sectional retrospective chart review of neonates of hypothyroid pregnant patients delivered at the Aga Khan University Hospital. Data was collected by trained medical students. It was randomly double-checked and corroborated by the principal investigator. We reviewed the medical record files of neonates of the mothers who were either a known case of hypothyroidism (overt as well as subclinical) or were diagnosed during their antenatal visits, during the year 2008–2016. We determined the timing of diagnosis of hypothyroidism (newly diagnosed as well as previously diagnosed) through data recorded by their primary physicians (endocrinologist or obstetricians) in relation to the gestation during the study period. We started the file review of the most recent case. We noted the data from neonatal files including neonatal TSH level of 2nd or 3rd day of life (whichever available).

We selected neonates born to mothers who were diagnosed hypothyroid according to the following inclusion criteria: All pregnant women attending endocrine and obstetric clinic who had been diagnosed overt [thyrotropin (TSH) > 2.5 mIU/l and low free tetra-iodothyronine (FT4) OR TSH > 10 mIU/l only] or subclinical (TSH 2.5 − 10 mIU/l and normal FT4) hypothyroidism either before or during pregnancy. Patients were either on levothyroxine replacement or newly started according to their presentation. Maternal characteristics and maternal outcomes of these neonates have already been published in a separate article (MHPO-1) [22]. Pregnancies with any abnormal thyroid function tests that did not fit into the overt or subclinical hypothyroidism criteria by American Thyroid Association were excluded from the study [23].

### Definitions of outcomes and congenital anomalies

#### Gestational age at birth (weeks):

We divided the gestational age at birth into “extremely preterm (<28)”, “very preterm (28–31)”, “late preterm (32–36)”, “term (37–42)” and “post-term (>42)”. ***Birth Weight:*** Defined as “Low (<2500 g)”, “Appropriate (2500–4000 g)” and “Large (>4000 g)”. ***Low APGAR score at 5 min:*** Assessment of physical health is noted in the one-minute APGAR score (scored from 0 to 10) and then again at five minutes to assess how the baby has responded to any resuscitation attempts. A score below 7 is considered low at 5 min. ***Respiratory complications*** (including respiratory distress syndrome, neonatal sepsis, and need for phototherapy): As indicated by the physician’s notes. ***Neonatal jaundice:*** Total bilirubin levels above 17 µmol/L checked within 48–72 h of birth. ***Hypocalcaemia:*** Serum calcium levels below the reference range defined for neonates in our hospital, that is, 146.2–173.4 µmol/L checked at any time after birth till hospital discharge. ***NICU (neonatal intensive care unit) admission:*** Neonates requiring ICU admission for 24 h or more. ***Neonatal death:*** Classified further as *early*; if occurs in less than 7 days or *late*; if occurs between 7 and 28 days of life. ***Congenital anomalies*** are defined using International Classification of Diseases (ICD-10) codes Q00-Q99 but excludes “inborn errors of metabolism” (codes E70-E90). Conditions of clinical importance like cardiovascular defects, nervous system defects and urogenital anomalies were grouped separately. Rest of the conditions of less clinical importance were also noted and detailed in the results.

### Statistical analysis

The results of demographic and clinical features are presented as mean ± standard deviation for quantitative variables and number (percentage) for qualitative variables. Proportional differences were assessed by using the Chi-square test or Fisher exact test where appropriate. For the purpose of subgroup analysis, variables of birth weight and gestational age at delivery were recoded dichotomously into low birth weight and premature birth. Unadjusted Odds ratio were calculated for the effect of timing of diagnosis of hypothyroidism on various neonatal outcomes and congenital anomalies. We sought ethical approval from the university’s ethical review committee (ERC number: 3977-Med-ERC-15).

## Results

**Figure 1.**
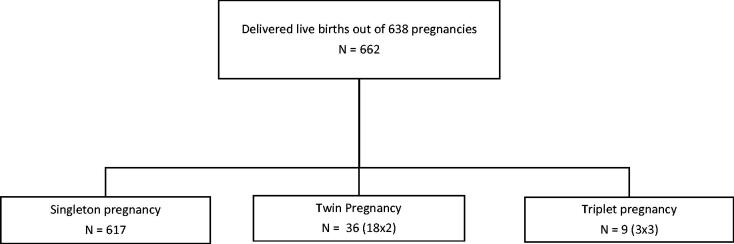
Flow chart for live births. Number of singleton, twin and triplet pregnancies.

Among 708 hypothyroid women recruited, 638 had live births (Figure 1). The mean age of the hypothyroid pregnancies was 31 years (SD 4.73). In our hospital, there was variation in the timing of presentation of women with respect to their gestational ages; however, almost two-third had presented in the first trimester, with most visits noted in the 8th week. Most of the patients were already diagnosed before pregnancy and subclinical hypothyroid cases were dominating in the overall population. The serum TSH levels were recorded in 53.2% and 56.7% cases in the preconception and 1st trimester, whereas 61.7% and 66.6% of cases had their TSH levels available in the second and third trimester periods.

**Figure 2.**
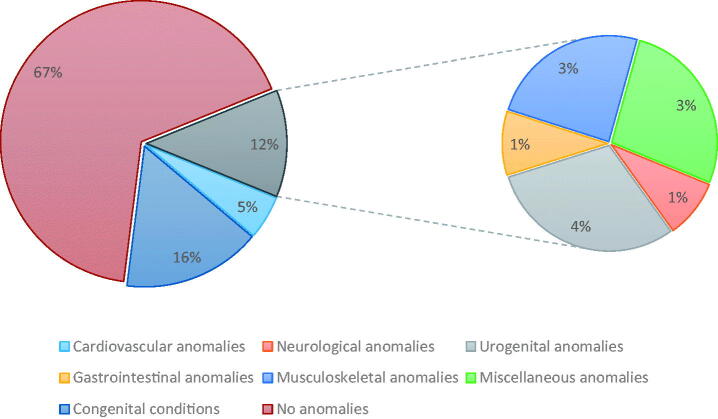
Major groups of congenital anomalies and conditions of neonates of hypothyroid women.

### Neonatal outcomes

Table 1 describes the detailed characteristics of neonatal outcomes. The mean gestational age at delivery was 37.5 weeks (SD 2.1). Neonatal jaundice was the most frequent medical condition (37.6%), measured on day 1–3 of life, with nearly half of the neonates having total bilirubin levels between 85.0 and 170.0 µmol/L. Single session phototherapy was required in almost 15% of neonates (Supplementary File, Table 1). Neonates with total bilirubin <255 µmol/L, requiring phototherapy were total 82 out of overall 98. Out of these, 25 had appropriate birth weight and born at term and 7 had low birth weight at term. Only one neonate had large birth weight at term whose mother had diabetes as well as gestational hypertension. Now out of those with appropriate birth weight at term, 1 had Glucose-6-Phosphate dehydrogenase (G6PD) deficiency, 1 had Down's syndrome and high TSH of 34.3 mIU/l. Eight mothers of these neonates had gestational diabetes (GDM), 3 had diabetes mellitus (DM), 3 had GH/preeclampsia and 2 had chronic hypertension. One mother also had past obstetric history of Trisomy 18. Since none of our neonates were diagnosed with congenital hypothyroidism in the short-term (within a month), we did not analyse the association of this level of hyperbilirubinemia with neonatal TSH levels. Although majority of the respiratory complications were not detailed in the charts, transient tachypnoea of newborn accounted for the most (0.9%) of them, and only three cases (0.5%) required ventilatory support. Sepsis was found amongst 6.5% of the neonates, out of which 1.2% were presumed to have sepsis, with only one case each for bacterial pneumonia, acute gastroenteritis, urosepsis, and meningitis. Out of 662 cases, 12% required NICU admission, but neonatal death was 1.6%. Amongst our cohort (Supplementary File, Table 2), 92% of the neonates had birth weight appropriate for gestational age at term; however, 56% were small for gestational age at even late preterm. There were five cases of large for gestational age, with only one of them being born to a diabetic mother who also had gestational hypertension. The median TSH of the neonates was 4.18 mIU/l at 48 h of birth and only nine neonates had a TSH of more than 20 mIU/l. The highest TSH recorded was 57.6 mIU/l.

**Table 1.** Characteristics of neonatal outcomes of hypothyroid mothers.

Neonatal outcomes	F (%)* N = 662
Gestational age at delivery (weeks)	
Extremely preterm (<28)	1 (0.2)
Very preterm (28–31)	14 (2.1)
Late preterm (32–36)	121 (18.3)
Term (37–42)	526 (79.5)
Birth weight (g)	
Low (<2500)	142 (21.5)
Appropriate (2500–4000)	512 (77.3)
Large (>4000)	5 (0.8)
Not documented	3 (0.5)
Respiratory complications	53 (8.0)
Sepsis	43 (6.5)
Hypocalcaemia	34 (5.1)
Neonatal Jaundice	66 (10.0)
Total Bilirubin 1.0–5.0 (mg/dl)	303 (45.8)
Total Bilirubin 5.1–10.0	199 (30.1)
Total Bilirubin 10.1–15.0	33 (5.0)
Total Bilirubin 15.1–20.0	61 (9.2)
Total Bilirubin not available	
Need for phototherapy	98 (14.8)
APGAR at 1 min	7.9 (± 0.6)
APGAR at 5 min	8.9 (± 0.4)
Low APGAR at 5 min (Scor*e* < 7)	3 (0.5)
NICU admission	
Less than 24 h	10 (1.5)
More than 24 h	70 (10.6)
Neonatal TSH (at 48 h) (mIU/L)	4.1 (2.4–6.8)
Neonatal death	
Early (<7days)	5 (0.8)
Late (between 7-28)	5 (0.8)

*Data are expressed as mean ± standard deviation, median (percentiles 25–75), or frequencies.

APGAR: Appearance, Pulse, Grimace, Activity, and Respiration; NICU: neonatal intensive care admission; TSH: thyroid-stimulating hormone.

### Congenital anomalies

Twenty-two percent (147/662) of the neonates had some forms of congenital anomalies, defects and conditions (Figure 2) (Supplementary File, Table 3). So, major congenital anomalies were classified into cardiovascular defects, nervous system defects and urogenital anomalies and accounted for 10.3% only. Overall, 48 neonates had two congenital defects and nine of 662 had three defects. Mongolian spots were the commonest congenital condition, but cardiovascular defects were the highest clinically significant congenital anomaly. Out of two cases of Down syndrome, one had a TSH of 34.3 uIU/L and the other had 11.0 uIU/L. One child was found to have learning difficulty at 6.5 years of age.

### Subgroup analysis of neonatal outcomes based on timing of diagnosis of hypothyroidism

Pregnant women in our cohort were mostly diagnosed with hypothyroidism prior to pregnancy (*N* = 565, 85.3%) with only 29 (4.4%) cases having no record. Upon subgroup analysis (Table 2), women diagnosed before conception had significant association with low birth weight and congenital anomalies in the neonates. However, there was only marginally significant association of premature birth and sepsis in the neonates, with respect to these two groups. Other neonatal outcomes had no clinically significant association with respect to the timings of diagnosis of hypothyroidism in pregnant women.

**Table 2.** Subgroup analysis of neonatal outcomes and congenital anomalies based on timing of diagnosis of hypothyroidism.

Neonatal outcomes	Diagnosis of maternal hypothyroidism		*p* Value	Odds ratio	95% CI^$^
	During pregnancy *N* = 68	Prior to pregnancy *N* = 565			
Low APGAR at 5 min					
Yes	0 (0.0%)	3 (0.5%)	1.000*	1.12	1.09–1.15
No	68 (100.0%)	560 (99.5%)			
Premature birth					
Yes	20 (29.4%)	108 (19.1%)	.055	1.76	1.00–3.09
No	48 (70.6%)	457 (80.9%)			
Low Birth Weight					
Yes	21 (31.3%)	111 (19.6%)	.028	1.86	1.07–3.25
No	46 (68.7%)	454 (80.4%)			
Respiratory distress syndrome					
Yes	7 (10.3%)	42 (7.4%)	.468	1.43	0.61–3.32
No	61 (89.7%)	523 (92.6%)			
Sepsis					
Yes	8 (11.8%)	31 (5.5%)	.057	2.29	1.01–5.22
No	60 (88.2%)	534 (94.5%)			
Hyperbilirubinemia					
Yes	22 (32.4%)	213 (37.7%)	.427	0.79	0.46–1.35
No	46 (67.6%)	352 (62.3%)			
Need for Phototherapy					
Yes	7 (10.3%)	85 (15.0%)	.364	0.65	0.28–1.46
No	61 (89.7%)	480 (85.0%)			
Hypocalcaemia					
Yes	5 (7.4%)	26 (4.6%)	.365	1.64	0.61–4.43
No	63 (92.6%)	538 (95.4%)			
NICU admission less than 24 h					
Yes	0 (0.0%)	10 (1.8%)	.611*	1.12	1.09–1.15
No	68 (100.0%)	555 (98.2%)			
NICU admission more than 24 h					
Yes	11 (16.2%)	55 (9.7%)	.137	1.78	0.88–3.61
No	57 (83.8%)	510 (90.3%)			
Neonatal death					
Yes	1 (1.5%)	8 (1.4%)	.643*	1.03	0.13–8.43
No	67 (98.5%)	557 (98.6%)			
Neonatal TSH groups (*N* = 588)^#^					
<20 mIU/l	59 (96.7%)	522 (98.8%)	.158*	0.28	0.05–1.48
≥20 mIU/l	2 (3.3%)	5 (1.2%)			
Congenital anomalies and conditions					
Yes	26 (38.2%)	116 (20.5%)	.001	2.39	1.41–4.07
No	42 (61.8%)	449 (79.5%)			

*p*-value determined by chi square. p value ≤0.05 taken as significant. *Fischer test applied. ^#^Neonatal TSH were missing in 74 cases. ^$^Unadjusted odds ration was calculated.

APGAR: Appearance, Pulse, Grimace, Activity, and Respiration; NICU: neonatal intensive care admission; TSH: thyroid-stimulating hormone.

## Discussion

Maternal hypothyroidism has been associated with adverse neonatal outcomes in various studies [14,24]. Neonatal hyperbilirubinemia is reported to be a common condition caused by variety of factors in Asians [25]. Studies from our city Karachi have also reported 11–13% prevalence of neonatal jaundice in general population [26,27]. Our babies had varying degrees of jaundice in the first month after birth as reported by a recent study conducted in India [24], but none of them was labelled to have congenital hypothyroidism, which is one of the rare but overlooked cause of severe and prolonged jaundice as reported in the literature [28]. Out of three cases of G6PD deficiency reported in our neonates, two had hyperbilirubinemia, and their TSH levels were not above the normal laboratory range (1.0–39.0 mIU/l). Besides, there was no reported case of ABO or Rh (Rhesus) incompatibility case in our cohort. Overall, these neonates should be followed, either for the resolution or worsening of jaundice, through a well-defined protocol in hypothyroid pregnancies.

Neonates of hypothyroid mothers in our hospital had similar rate (12%) of NICU admission, as described in the international literature. A recent Finnish study reported 14.4% NICU admissions in a large cohort of 16,364 hypothyroid mothers which was only slightly higher (13%) than those mothers who were consistently using levothyroxine replacement amongst them (OR 1.09, CI 1.01–1.18) [18]. Similarly, a Nigerian study also reported 16% NICU admission in babies of hypothyroid mothers which was significantly higher than babies of normal mothers (6.3%, *p* ≤0.05), although the sample size was relatively small [29]. We had prolonged NICU stay (>24 h) in 10.6% of our neonates and only 1.5% required <24 h NICU care. Although, prospective case–control studies are required to further elaborate on factors responsible for this prevalence of NICU admissions in our setup, neonates of hypothyroid mothers are generally reported to require increased NICU care than euthyroid mothers [30]. Overall, only 1.6% neonatal deaths occurred in our cohort, which is considerably lower as compared to 4.2–13% as reported in local studies [31,32].

A recent cross-sectional study from Bangladesh has reported maternal hypothyroidism to be present in 5.12% of babies born with congenital anomalies [17]. Studies from Pakistan have reported 5–7% prevalence of congenital anomalies overall [33,34]. Several studies are reported about the association of congenital maternal hypothyroidism and the risk of neurodevelopment impairment of their children [35,36]. However, data are still conflicting about this strong association in mothers without congenital hypothyroidism; hence, it is difficult to report this from the literature [19–21]. Majority of the mothers in our cohort had Hashimoto’s disease as the aetiology of hypothyroidism, however, only one patient had congenital hypothyroidism with no congenital anomaly in her neonate. Our mothers had their TSH levels aimed to be controlled below 2.5 mIU/l with varying requirement of levothyroxine dose (results to be published in a separate paper, MHPO-5) [22]. Besides congenital anomalies are more evidently reported in co-existence with neonatal congenital hypothyroidism [37,38]. Only nine neonates in our study group had TSH raised more than 20 mIU/l and none of them developed congenital hypothyroidism on follow-up up to 1 month. We therefore compared our data with congenital hypothyroid neonates. Neonates with Down syndrome are also found to have congenital hypothyroidism along with other thyroid disorders [39,40]. We had only two cases of Down syndrome and only one had TSH > 20 mIU/l, but there was no hypothyroidism on follow-up up to 1 month. Another unique observation in our study is the higher chance (OR 2.39, 95% CI 1.4–4.0, *p* ≤0.05) of congenital anomalies in the neonates of women diagnosed hypothyroid prior to pregnancy as compared to those diagnosed during pregnancy. A recent Spanish study showed 53.7% women were diagnosed hypothyroidism before pregnancy [41], and another large nationwide study from Finland reported perinatal and pregnancy outcomes in hypothyroid women who had pregestational (65.9%) as well as gestational (37.5%) diagnosis based on levothyroxine purchases [18]. But none of them compared the outcomes between these two groups as reported in our study.

Most studies reported major congenital anomalies as a perinatal outcome of hypothyroid mothers but did not describe the details of the anomalies [18]. Among the significant congenital anomalies, our cohort had more cardiovascular defects (CVD) with highest prevalence of Patent Ductus Arteriosus (PDA) (1.2%) followed by Ventricular Septal Defect (VSD) (1.1%). Literature is limited regarding neonates of hypothyroid mothers without congenital hypothyroidism developing cardiovascular defects. A study from India reported higher CVD than other anomalies amongst congenital hypothyroid neonates [42], which is similar to a Mexican study with a larger sample size and with PDA as the second most common CVD [43]. Urogenital tract was the second clinically important organ system involved in our neonates. A study from Iran has reported significant association of presence of urogenital anomalies with congenital hypothyroidism (OR 2.04; 95%CI: 1.1–3.6; *p* ≤0.05) [38]. We also observed that births to women diagnosed during pregnancy are 2.3 times more likely to have a congenital anomaly or condition. We have no local data to compare this effect, however, this subject is also rarely explored in literature. One of our neonates also had Zellweger syndrome and the other had Edward syndrome, which have never been reported before. Although there are number of miscellaneous cutaneous (most common Mongolian spots) and musculoskeletal conditions present in our cohort of neonates, we need to identify their significance only after conducting a case–control study.

Neonates in our cohort had significant association with low birth weight (OR 1.86, 95% CI 1.0–3.2, *p* ≤0.05) in hypothyroid women diagnosed before pregnancy. This is in contrast to a large Danish registry, which reported increased risk of high birth weight associated with hypothyroidism in mothers (adjusted difference 20 g, 95% CI 10–30 g) rather than low birth weight which was actually associated with hyperthyroidism, even after adjusting for potential confounders [44]. On the other hand, many studies have concluded that maternal hypothyroidism had no significant association with low birth weight in early as well as late pregnancy [45,46]. However, other studies described lower birth weight in the hypothyroid women as compared to control groups (*p* ≤ 0.05) as reported in our study [47]. However, no study has directly compared this outcome with timing of maternal diagnosis of hypothyroidism, which is the novelty described in our study.

Our study also observed marginally significant association of premature birth (OR 1.76, CI 1.00–3.09, *p* ≤ 0.05) and sepsis (OR 2.29, CI 1.01–5.22, *p* ≤ 0.05) with the timing of diagnosis of maternal hypothyroidism, with higher chance in those diagnosed prior to pregnancy. Premature birth is a known complication of maternal hypothyroidism as reported in many studies [18,48,49], however, there are studies which have also shown no such association [45]. Differences in opinion may be due to heterogenous population and differences in study design, most of the data being retrospectively collected or retrieved from birth registries. Similarly, neonatal sepsis has been well reported in association with maternal hypothyroidism (a OR 1.42, 95% CI 1.16–1.74) [30]. When reviewing these studies in detail, none have compared these outcomes with respect to the timing of diagnosis of maternal hypothyroidism.

In conclusion, our study described hyperbilirubinaemia as the most common neonatal outcome and cardiovascular defects as the most common major congenital anomaly. There is significant association of congenital anomalies and low birth weight with the timing of diagnosis of hypothyroidism in women. Moreover, premature birth and neonatal sepsis is also associated in this respect with marginal significance. Our study also has a limitation with regards to selection bias. We observed higher congenital anomalies and conditions in neonates born to women diagnosed during pregnancy. As this is a single hospital study, women with known hypothyroidism could have been more likely referred into our hospital for the high-risk obstetrical care with lessor congenital defects. Despite of all the limitations associated with a retrospective study design, this is the first study reporting neonatal outcomes and congenital anomalies of hypothyroid pregnancies from Pakistan. We propose to do multi-centre prospective studies to determine the association of congenital anomalies in neonates of uncontrolled hypothyroid mothers either diagnosed before or during pregnancy with adequate levothyroxine replacement. We also recommend to perform case–control studies to determine the effects of hypothyroid pregnancies on the neonatal outcomes, whether diagnosed before pregnancy or anytime during gestation.

## Supplementary Material

Supplemental Material

## Data Availability

The dataset supporting the findings of this study can be made available upon request to the first author whose email is drzareenkiran@gmail.com.

## References

[1] Azizi F, Delshad H. Thyroid derangements in pregnancy. Iran J Endocrinol Metabol. 2014;15(6):491–508.

[2] Wilson KL, Casey BM, McIntire DD, et al. Subclinical thyroid disease and the incidence of hypertension in pregnancy. Obstet Gynecol. 2012;119(2 Pt 1):315–320. 22270283 10.1097/AOG.0b013e318240de6a

[3] Vanes NK, Charlesworth D, Imtiaz R, et al. Optimal treatment of hypothyroidism associated with live birth in cases of previous recurrent placental abruption and stillbirth. Int J Gynaecol Obstet. 2013;123(3):196–199. 24055168 10.1016/j.ijgo.2013.05.025

[4] Cao XY, Jiang XM, Dou ZH, et al. Timing of vulnerability of the brain to iodine deficiency in endemic cretinism. N Engl J Med. 1994;331(26):1739–1744. 7984194 10.1056/NEJM199412293312603

[5] Davis LE, Leveno KJ, Cunningham FG. Hypothyroidism complicating pregnancy. Obstet Gynecol. 1988;72(1):108–112. 3380497

[6] Casey BM, Dashe JS, Wells CE, et al. Subclinical hypothyroidism and pregnancy outcomes. Obstet Gynecol. 2005;105(2):239–245. 15684146 10.1097/01.AOG.0000152345.99421.22

[7] Pop VJ, Brouwers EP, Vader HL, et al. Maternal hypothyroxinaemia during early pregnancy and subsequent child development: a 3-year follow-up study. Clin Endocrinol. 2003;59(3):282–288. 10.1046/j.1365-2265.2003.01822.x 12919150

[8] Ahmed OM, El-Gareib AW, El-Bakry AM, et al. IJoDN. Thyroid hormones states and brain development interactions. Int J Dev Neurosci. 2008;26(2):147–209. 18031969 10.1016/j.ijdevneu.2007.09.011

[9] Haddow JE, Palomaki GE, Allan WC, et al. Maternal thyroid deficiency during pregnancy and subsequent neuropsychological development of the child. N Engl J Med. 1999;341(8):549–555. 10451459 10.1056/NEJM199908193410801

[10] Morreale de Escobar G, Obregón MJ, Escobar del Rey F. JTJoce, metabolism. Is neuropsychological development related to maternal hypothyroidism or to maternal hypothyroxinemia? J Clin Endocrinol Metab. 2000;85(11):3975–3987. 11095417 10.1210/jcem.85.11.6961

[11] Matsuura N, Konishi J. JEj. Transient hypothyroidism in infants born to mothers with chronic thyroiditis-a nationwide study of twenty-three cases. The Transient Hypothyroidism Study Group. Endocrinol JPN. 1990;37(3):369–379. 2253587 10.1507/endocrj1954.37.369

[12] Woeber KA. Subclinical thyroid dysfunction. Arch Intern Med. 1997;157(10):1065–1068. 9164371

[13] Canaris GJ, Manowitz NR, Mayor G, et al. The Colorado thyroid disease prevalence study. Arch Intern Med. 2000;160(4):526–534. 10695693 10.1001/archinte.160.4.526

[14] Liu Y, Chen H, Jing C, et al. The association between maternal subclinical hypothyroidism and growth, development, and childhood intelligence: a meta-analysis. Jcrpe. 2018;10(2):153–161. 28958983 10.4274/jcrpe.4931 PMC5985385

[15] Barber K, Franklyn J, McCabe C, et al. The in vitro effects of triiodothyronine on epidermal growth factor-induced trophoblast function. J Clin Endocrinol Metab. 2005;90(3):1655–1661. 15598697 10.1210/jc.2004-0785

[16] Stagnaro-Green A, Chen X, Bogden JD, et al. The thyroid and pregnancy: a novel risk factor for very preterm delivery. Thyroid. 2005;15(4):351–357. 15876159 10.1089/thy.2005.15.351

[17] Fatema K, Das T, Mannan A, et al. Frequency, distribution of congenital anomaly and associated maternal risk factors. MMJ. 2017;26(3):658–666. 28919624

[18] Turunen S, Vaarasmaki M, Mannisto T, et al. Pregnancy and perinatal outcome among hypothyroid mothers: a population-based cohort study. Thyroid. 2019;29(1):135–141. 30417761 10.1089/thy.2018.0311

[19] Nazarpour S, Ramezani Tehrani F, Simbar M, et al. Effects of levothyroxine treatment on pregnancy outcomes in pregnant women with autoimmune thyroid disease. Eur J Endocrinol. 2017;176(2):253–265. 27879326 10.1530/EJE-16-0548

[20] Nazarpour S, Ramezani Tehrani F, Simbar M, et al. Effects of levothyroxine on pregnant women with subclinical hypothyroidism, negative for thyroid peroxidase antibodies. J Clin Endocrinol Metab. 2018;103(3):926–935. 29126290 10.1210/jc.2017-01850

[21] Casey BM, Thom EA, Peaceman AM, et al. Treatment of subclinical hypothyroidism or hypothyroxinemia in pregnancy. N Engl J Med. 2017;376(9):815–825. 28249134 10.1056/NEJMoa1606205 PMC5605129

[22] Kiran Z, Sheikh A, Malik S, et al. Maternal characteristics and outcomes affected by hypothyroidism during pregnancy (maternal hypothyroidism on pregnancy outcomes, MHPO-1). BMC Pregnancy Childbirth. 2019;19(1):476. 31805890 10.1186/s12884-019-2596-9 PMC6896307

[23] Alexander EK, Pearce EN, Brent GA, et al. 2017 Guidelines of the american thyroid association for the diagnosis and management of thyroid disease during pregnancy and the postpartum. Thyroid. 2017;27(3):315–389. 28056690 10.1089/thy.2016.0457

[24] Al Nemri AM, Al-Jurayyan RN, Mohamed S, et al. Neonatal hyperbilirubinemia and elevated liver enzymes associated with thyroid hormone deficiency in neonates. Curr Pediatr Res. 2012.

[25] Odum EP, Orazulike NC. Neonatal outcomes of mothers with hypothyroidism in the third trimester. Development. 2016;3:4.

[26] Mannisto T, Mendola P, Reddy U, et al. Neonatal outcomes and birth weight in pregnancies complicated by maternal thyroid disease. Am J Epidemiol. 2013;178(5):731–740. 23666815 10.1093/aje/kwt031 PMC3755642

[27] Ali SM, Karamat S, Khawaja AAJ, et al. Frequency of maternal and neonatal complications associated with second stage caesarean section in civil hospital karachi. TPMJ. 2020;27(03):535–539.

[28] Habib MA, Black KI, Ariff S, et al. Effect of kangaroo mother care on neonatal health outcomes in rural Pakistan, a randomized controlled trial. 2019;2:1–24.

[29] Gustavson KH. Prevalence and aetiology of congenital birth defects, infant mortality and mental retardation in lahore, Pakistan: a prospective cohort study. Acta Paediatr. 2005;94(6):769–774. 16188785 10.1111/j.1651-2227.2005.tb01981.x

[30] Hussain S, Asghar I, Sabir M, et al. Prevalence and pattern of congenital malformations among neonates in the neonatal unit of a teaching hospital. J Pak Med Assoc. 2014;64(6):629–634. 25252479

[31] Leger J, Forhan A, Dos Santos S, et al. Developmental milestones at one year for the offspring of mothers with congenital hypothyroidism: a population-based study. Eur J Endocrinol. 2018;178(5):471–480. 29500308 10.1530/EJE-17-0855

[32] Clairman H, Skocic J, Lischinsky JE, et al. Do children with congenital hypothyroidism exhibit abnormal cortical morphology? Pediatr Res. 2015;78(3):286–297. 25978801 10.1038/pr.2015.93

[33] Peters C, van Trotsenburg ASP, Schoenmakers N. Diagnosis of endocrine disease: congenital hypothyroidism: update and perspectives. Eur J Endocrinol. 2018;179(6):R297–R317. 30324792 10.1530/EJE-18-0383

[34] Yousefi Chaijan P, Dorreh F, Sharafkhah M, et al. Congenital urogenital abnormalities in children with congenital hypothyroidism. Med J Islam Repub Iran. 2017;31:7. 28638814 10.18869/mjiri.31.7 PMC5473016

[35] Amr NH. Thyroid disorders in subjects with down syndrome: an update. Acta Bio-Medica: Atenei Parmensis. 2018;89(1):132–139. 29633736 10.23750/abm.v89i1.7120 PMC6357620

[36] Pierce MJ, LaFranchi SH, Pinter JD. Characterization of thyroid abnormalities in a large cohort of children with Down syndrome. Horm Res Paediatr. 2017;87(3):170–178. 28259872 10.1159/000457952 PMC5483988

[37] Donnay S, Fajardo C, Fernández-García JC, et al. Diagnosis, treatment, and management of gestational hypothyroidism. The Tirogest Study. 2020;67(1):36–42. 10.1016/j.endinu.2019.03.008 31109823

[38] Konar H, Sarkar M, Roy M. Association of thyroid dysfunction and autoimmunity in pregnant women with diabetes mellitus. J Obstet Gynaecol India. 2018;68(4):283–288. 30065543 10.1007/s13224-017-1033-0 PMC6046673

[39] Huang M-J, Kua K-E, Teng H-C, et al. Risk factors for severe hyperbilirubinemia in neonates. Pediatr Res. 2004;56(5):682–689. 15319464 10.1203/01.PDR.0000141846.37253.AF

[40] Parkash J, Das N. Pattern of admissions to neonatal unit. J Coll Physicians Surg Pak. 2005;15(6):341–344. 15924838

[41] Arif M. Neonatal jaundice due to rhesus and AB isoimmunization and frequency of these antigens in pakistani mothers. JPMA. 1982;2:165–167. 6813526

[42] Reddy PA, Rajagopal G, Harinarayan CV, et al. High prevalence of associated birth defects in congenital hypothyroidism. Int J Pediatr Endocrinol. 2010;2010:940980. 20454578 10.1155/2010/940980 PMC2864451

[43] Monroy-Santoyo S, Ibarra-Gonzalez I, Fernandez-Lainez C, et al. Higher incidence of thyroid agenesis in mexican newborns with congenital hypothyroidism associated with birth defects. Early Hum Dev. 2012;88(1):61–64. 21816548 10.1016/j.earlhumdev.2011.07.009

[44] Andersen SL, Olsen J, Wu CS, Laurberg P. Low birth weight in children born to mothers with hyperthyroidism and high birth weight in hypothyroidism, whereas preterm birth is common in both conditions: a danish national hospital register study. Eur Thyroid J. 2013;2(2):135–144. 24783052 10.1159/000350513 PMC3821508

[45] Wang S, Teng WP, Li JX, et al. Effects of maternal subclinical hypothyroidism on obstetrical outcomes during early pregnancy. J Endocrinol Invest. 2012;35(3):322–325. 21642766 10.3275/7772

[46] Idris I, Srinivasan R, Simm A, et al. Maternal hypothyroidism in early and late gestation: effects on neonatal and obstetric outcome. Clin Endocrinol. 2005;63(5):560–565. 10.1111/j.1365-2265.2005.02382.x 16268809

[47] Blazer S, Moreh-Waterman Y, Miller-Lotan R, et al. Maternal hypothyroidism may affect fetal growth and neonatal thyroid function. Obstet Gynecol. 2003;102(2):232–241. 12907094 10.1016/s0029-7844(03)00513-1

[48] Nasirkandy MP, Badfar G, Shohani M, et al. The relation of maternal hypothyroidism and hypothyroxinemia during pregnancy on preterm birth: an updated systematic review and Meta-analysis. Int J Reprod Biomed. 2017;15(9):543. 29662962 PMC5894470

[49] Mansouri A, Norouzi S, Sharifi A, et al. The relationship of maternal subclinical hypothyroidism during pregnancy and preterm birth: a systematic review and Meta-analysis of cohort studies. J Clin Endocrinol Metab. 2017;19(40):69–78.

