# Supplementary material for: Neonatal outcomes and congenital anomalies in pregnancies affected by hypothyroidism
Source: Ann Med. 2021 Sep 8;53(1):1560–8. doi: 10.1080/07853890.2021.1970798 (PMC8439228; doi:10.1080/07853890.2021.1970798)
Supplement: Supplemental Material [file IANN_A_1970798_SM8511.docx]

TABLE 1- NEONATAL JAUNDICE AND NEED FOR PHOTOTHERAPY

| **Bilirubin levels in neonatal jaundice (µmol/L)** | **Number of neonates who needed phototherapy** | |
| --- | --- | --- |
|  | **yes** | **no** |
| Total Bilirubin 17.10-85.52 | 11 (16.7%) | 55 (83.3%) |
| Total Bilirubin 85.52-171.04 | 26 (8.6%) | 277 (91.4%) |
| Total Bilirubin 171.04-256.56 | 45 (22.6%) | 154 (77.4%) |
| Total Bilirubin 256.56-342.08 | 16 (48.5%) | 17 (51.5%) |
| Total Bilirubin not available | 0 (0%) | 61 (100%) |

TABLE 2- BIRTH WEIGHTS ACROSS DIFFERENT GESTATIONAL AGE AT BIRTH OF NEONATES.

| **Gestational age at birth (weeks)** | **Birth Weight Groups (g)** | | |
| --- | --- | --- | --- |
|  | **Low (<2500 g)** | **Appropriate (2500-4000 g)** | **Large (>4000 g)** |
| Extremely preterm (<28) | 1 (0.7%) | 0 (0%) | 0 (0%) |
| Very preterm (28-31) | 14 (9.9%) | 0 (0%) | 0 (0%) |
| Late preterm (32-36) | 80 (56.3%) | 41 (8.0%) | 0 (0%) |
| Term (37-42) | 47 (33.1%) | 471 (92.0%) | 5 (100.0%) |

Abbreviations: g, grams.

TABLE 3. CONGENITAL ANOMALIES AND CONDITIONS OF NEONATES OF HYPOTHYROID WOMEN.

| **CONGENITAL ANOMALIES** | **Frequency (%)** |
| --- | --- |
| **Cardiovascular anomalies**  Patent Ductus Arteriosus  Ventricular Septal Defect  Atrial Septal Defect  Congenital Heart Disease  Patent Foramen Ovale  Coarctation of aorta  Localized hypertrophy of apical Interventricular Septum  Left ventricular hypertrophy and dilatation  Right ventricular hypertrophy  Systolic murmur | **n = 31**  8 (1.2)  7 (1.1)  5 (0.8)  4 (0.6)  2 (0.3)  1 (0.2)  1 (0.2)  1 (0.2)  1 (0.2)  1 (0.2) |
| **Neurological anomalies**  Cerebellar hypoplasia  Arnold Chiari malformation  Hydrocephalus  Meningomyelocoele  Lisencephaly | **n= 6**  2 (0.3)  1 (0.2)  1 (0.2)  1 (0.2)  1 (0.2) |
| **Urogenital anomalies**  Renal anomaly  Antenatal hydronephrosis of left side  Unilateral undescended testis  Bilateral undescended testis  Small hydrocele on right side  Small testis of left side  Retractile testis of left side  Bilateral retractile testis  Congenital Nephrotic syndrome  Ambiguous genitalia  Hypospadias  Chordae penis | **n = 20**  3 (0.5)  3 (0.5)  3 (0.6)  2 (0.3)  2 (0.4)  1 (0.2)  1 (0.2)  1 (0.2)  1 (0.2)  1 (0.2)  1 (0.2)  1 (0.2) |
| **Gastrointestinal anomalies**  Intestinal obstruction  Malrotation of gut  Imperforate anus  Microcolon  Internal herniation of organ | **n = 6**  1 (0.2)  1 (0.2)  2 (0.4)  1 (0.2)  1 (0.2) |
| **Musculoskeletal anomalies**  Polydactyly  Bilateral talipes equinovarus  Cleft lip & palate  Micrognathia  Diaphragmatic hernia  Pre-axial pedunculated polydactyly over right thumb  Bilateral Rocker bottom feet  Deviated Nose  Fracture humerus  Gingival hyperplasia  Disproportionate pelvis | **n= 16**  3 (0.5)  2 (0.3)  2 (0.4)  2 (0.4)  1 (0.2)  1 (0.2)  1 (0.2)  1 (0.2)  1 (0.2)  1 (0.2)  1 (0.2) |
| **Miscellaneous**  Asymmetrical IUGR  G6PD deficiency  Dysmorphic features  Down's syndrome  Hyperglycemia  Natal teeth  Hypoglycemia  Edward syndrome  Zellweger syndrome  Thrombocytopenia | **n = 19**  4 (0.7)  3 (0.5)  2 (0.3)  2 (0.3)  2 (0.4)  2 (0.3)  1 (0.2)  1 (0.2)  1 (0.2)  1 (0.2) |
| **CONGENITAL CONDITIONS** | **Frequency (%)** |
| **Cutaneous (ectodermal) conditions**  Mongolian spots  Sacral dimple  Plethoric skin  Tuft of hair  Erythema toxicum neonatorum  Congenital pigmentary skin  Left ear tubercle  Hemangioma on eyes  Extra hair growth on ear  Skin tag on scrotum  Hemangioma on neck | **n = 78**  50 (7.6)  14 (2.2)  3 (0.5)  3 (0.5)  2 (0.4)  1 (0.2)  1 (0.2)  1 (0.2)  1 (0.2)  1 (0.2)  1 (0.2) |
| **Miscellaneous**  Positional talipes  Vaginal tag  Tongue tie  Epstein pearl near urethral meatus | **n = 22**  11 (1.7)  7 (1.1)  3 (0.5)  1 (0.2) |

Abbreviations: IUGR: intrauterine growth retardation, G6PD: Glucose-6-Phosphate Deficiency.
